# Molecular characterization of human respiratory syncytial virus in Seoul, South Korea, during 10 consecutive years, 2010–2019

**DOI:** 10.1371/journal.pone.0283873

**Published:** 2023-04-06

**Authors:** Ha Nui Kim, Jinha Hwang, Soo-Young Yoon, Chae Seung Lim, Yunjung Cho, Chang-Kyu Lee, Myung-Hyun Nam

**Affiliations:** Department of Laboratory Medicine, Korea University College of Medicine, Seoul, Korea; University of Pécs: Pecsi Tudomanyegyetem, HUNGARY

## Abstract

Respiratory syncytial virus (RSV) is the leading cause of lower respiratory tract infections and hospitalization in infants and young children. Here, we analyzed the genetic diversity of RSV using partial G gene sequences in 84 RSV-A and 78 RSV- B positive samples collected in Seoul, South Korea, for 10 consecutive years, from 2010 to 2019. Our phylogenetic analysis revealed that RSV-A strains were classified into either the ON1 (80.9%) or NA1 (19.0%) genotypes. On the other hand, RSV-B strains demonstrated diversified clusters within the BA genotype. Notably, some sequences designated as BA-SE, BA-SE1, and BA-DIS did not cluster with previously identified BA genotypes in the phylogenetic trees. Despite this, they did not meet the criteria for the assignment of a new genotype based on recent classification methods. Selection pressure analysis identified three positive selection sites (amino acid positions 273, 274, and 298) in RSV-A, and one possible positive selection site (amino acid position 296) in RSV-B, respectively. The mean evolutionary rates of Korean RSV-A from 1999 to 2019 and RSV-B strains from 1991 and 2019 were estimated at 3.51 × 10^−3^ nucleotides (nt) substitutions/site/year and 3.32 × 10^−3^ nt substitutions/site/year, respectively. The population dynamics in the Bayesian skyline plot revealed fluctuations corresponding to the emergence of dominant strains, including a switch of the dominant genotype from NA1 to ON1. Our study on time-scaled cumulative evolutionary analysis contributes to a better understanding of RSV epidemiology at the local level in South Korea.

## Introduction

Respiratory syncytial virus (RSV) is one of the most common causative organisms for bronchiolitis and pneumonia in infants and young children [1]. RSV infection affects over 60% of all children within their first year of life, and nearly all children are infected with RSV at least once within two years of birth, with recurrent RSV infections throughout lifetime [2, 3]. Thus, RSV-related hospital admissions result in a substantial burden on patients and healthcare systems [4].

RSV is an RNA-enveloped virus and a member of the *Pneumoviridae* family and has a non-segmented, negative-sense single-stranded viral RNA genome of approximately 15,000 nucleotides (nt) [5]. RSV is divided into two major subgroups, RSV-A and RSV-B, which diverged approximately 350 years ago based on their antigenic variations associated with attachment G glycoprotein [6, 7]. Specific genotypes within each RSV subgroup can be identified based on nucleotide variations within the second hypervariable region (2^nd^ HVR) of the G gene spanning amino acids 282–321 [8]. The categorization into specific genotypes is useful because the genetic variability of RSV is believed to be responsible for its ability to infect hosts repeatedly [9]. There are 13 genotypes (GA1-GA7, NA1-NA4, ON1, and SAA1) identified for RSV-A and at least 37 genotypes for RSV-B (GB1, GB2, GB3, GB4, GB5, GB6, GB12, GB13, SAB1, SAB2, SAB3, SAB4, URU1, URU2, CB1, THB, BA1, BA2, BA3, BA4, BA5, BA6, BA7, BA8, BA9, BA10, BA11, BA12, BA13, BA14, BA-C, BA-CCA, BA-CCB, JAB1, NZB1, and NZB2) so far worldwide [10, 11].

The 2^nd^ HVR of the G gene, which is highly variable and susceptible to selection pressure, is the most commonly utilized region for investigating the molecular epidemiology [12, 13]. Studying the molecular epidemiology and genetic evolution of RSV is essential for monitoring the emergence of new strains and for future vaccine development. There are currently 38 vaccine candidates for RSV under development, and 19 of them are undergoing clinical trials [14]. However, no RSV vaccine has been approved for release on the market, and treatment options remain limited. The need for continuous monitoring of RSV molecular epidemiology is increasing to ensure the effectiveness of developing vaccines and monoclonal antibodies.

The evolution of RSV is an ongoing dynamic process with the emergence of new genotypes and the disappearance of earlier ones. Previous studies of RSV in Korea have reported a generational shift from the NA1 to the ON1 genotype for RSV-A as of 2011, as well as a dominance of the BA9 and BA10 genotypes for RSV-B [1, 8, 15, 16]. In this context, we aimed to examine the molecular epidemiology of RSV for 10 consecutive years (2010–2019) in two hospitals in Seoul.

## Materials and methods

### Sample and data collection

We used the leftover nasopharyngeal swab specimens collected from patients who visited Korea University Hospital with signs of acute respiratory infection from 2010 to 2019. The multiplex RT-PCR method, Seeplex® RV15 One-Step ACE Detection assay or Anyplex™ II RV16 detection kit (Seegene, Inc., Seoul, South Korea), was used to detect the following respiratory viruses; adenovirus, coronavirus 229E/NL63, coronavirus OC43, parainfluenza virus 1, parainfluenza virus 2, parainfluenza virus 3, parainfluenza virus 4, influenza A virus, influenza B virus, human rhinovirus A/B/C, bocavirus 1/2/3/4, enterovirus, metapneumovirus, including RSV-A and RSV-B. In total, 244 samples, of which 122 were diagnosed as either RSV-A or RSV-B, were collected separately and stored at -70°C until further use. To analyze the occurrence patterns of RSV cases from 2010 to 2019, we investigated the number of RSV cases confirmed by either multiplex RT-PCR or culture using R-mix viral culture (Diagnostic Hybrids, Inc., Athens, OH, USA) with immunofluorescence. This study was approved by the Institutional Review Board (IRB) of the Korea University Guro Hospital (IRB number: 2022GR0052). The Ethics Committee waived the requirement of informed consent, as this study exclusively involved data on the isolated RSVs and the corresponding year of their acquisition.

### RT-PCR and sequencing for analysis of 2^nd^ HVR of G gene

The viral nucleic acids from the collected nasopharyngeal swab specimens were extracted using the LaboPass^TM^ Labozol reagent (CosmoGenetech, Seoul, South Korea)/STARMag 96 Virus kit (Seegene Inc., Seoul, Korea). The extracted RNA was reverse-transcribed into cDNA using a LaboPassTM cDNA synthesis kit (CosmoGenetech)/cDNA Synthesis Automix (Seegene). Target gene-specific primer pairs (5′–3′) were as follows: G151–173F: CTGGCAATGATAATCTCAACTTC, F3–22R: CAACTCCATTGTTATTTGC [17]. The PCR product was amplified using the following process: initial denaturation for 5 min at 95°C, 35 cycles of denaturation at 94°C for 30 s, annealing at 56°C for 30 s, extension at 72°C for 30 s, and a final extension step at 75°C for 5 min. The amplified products were separated by electrophoresis on 1–1.5% agarose gel and purified using a LaboPass™ Gel Extraction Kit (CosmoGenetech)/ Millipore plate MSNU030 (Millipore SAS, Molsheim, France). The purified PCR products were subjected to Sanger sequencing using a BigDye Terminator v3.1 sequencing kit and a 3730xl automated sequencer (Applied Biosystems, Foster City, CA).

### Phylogenetic analysis

Multiple sequence alignment using MUSCLE and further editing were performed using MEGA X [18]. Reference sequences representing each subtype corresponding to 13 subtypes of RSV-A (n = 111) and 36 subtypes of RSV-B (n = 185) were retrieved from GenBank by referring to relevant publications [10, 11, 16] (S1 and S2 Tables). Neighbor-joining (NJ) trees of RSV-A and RSV-B were constructed using the maximum composition likelihood model provided in MEGA X, followed by construction of maximum likelihood (ML) trees of RSV-A and RSV-B with the selected most suitable nucleotide substitution model, TN93 + G. Statistical significance of the tree topology was tested by bootstrapping in 1,000 replicates. Clusters with high statistical support were defined when a bootstrap value of ≥70% or a posterior probability of ≥0.8 [19, 20]. Only bootstrap values ≥70% are shown for the NJ and ML trees. The process of further genotype assignment was performed in accordance with protocols described in previous studies [10, 11]. Pairwise distances (*p*-distances) were calculated using MEGA X to compare the within and between group distances for each genotype.

Bayesian trees were inferred using Markov Chain Monte Carlo (MCMC) method implemented in BEAST v1.10.4 [21]. Time-scaled maximum clade credibility (MCC) trees were constructed under the relaxed clock log normal and coalescent exponential population prior model, selected based on marginal likelihood calculated by nested sampling [22]. The best-fit substitution model, GTR + F + G4 and TN + F + G4, were determined using ModelFinder for RSV-A and RSV-B, respectively [23]. The length of the MCMC chain was 500 million, and the samples were recorded at a frequency of 5,000. The tree was summarized and visualized using TreeAnnotator v1.10.4 and FigTree v1.4.4 (http://tree.bio.ed.ac.uk/software/figtree/) after a 10% burn-in. An acceptable effective sample size of ≥200 was confirmed using Tracer v1.7.2 (http://tree.bio.ed.ac.uk/software/tracer/). In terms of statistical support, posterior probabilities greater than 0.8 are considered well-supported [19].

### Analysis of amino acid sequence

The deduced amino acid sequences in the 2^nd^ HVR of the G gene were compared with the prototype strain of the RSV-A ON1 genotype (GenBank accession number JN257693) and the RSV-B BA prototype (GenBank accession number AY333364) after genotype assignment using phylogenetic trees. Mutations in the amino acid sequences were analyzed and visualized using Unipro UGENE [24].

#### Entropy analysis

The amino acid variations in the 2^nd^ HVR of the G gene were analyzed by Shannon entropy plot using BioEdit [25]. The sites with entropy values < 0.2 were considered conserved, and entropy values > 0.2 were considered variable [26].

#### Selection pressure analysis

The mutation rates of synonymous (*d*S) and nonsynonymous (*d*N) substitutions are used to assess whether sites in the G gene are subject to selective pressure. A *d*N /*d*S ratio >1 indicates positive selection, while a *d*N/*d*S ratio <1 indicates negative selection [27]. The 2^nd^ HVR of the G gene of RSV-A and RSV-B was analyzed with Datamonkey to estimate the selection pressure and identify codons under purifying or diversifying selection [28]. The *d*N and *d*S values for each codon were calculated using single likelihood ancestor counting (SLAC), fixed effects likelihood (FEL) [29], mixed effects model of evolution (MEME) [30], and fast, unbiased Bayesian approximation (FUBAR) [31].

### Virus effective population analysis

Bayesian skyline plot (BSP) was introduced to estimate the time-scaled evolutionary relationship for each RSV-A and RSV-B. The partial G gene sequences obtained in this study along with additional sequences retrieved from GenBank, consisting of 125 RSV-A and 67 RSV-B sequences previously reported in South Korea, were utilized for the estimation using BEAST v1.10.4. The appropriate substitution model was selected using the ModelFinder with TIM3 + F + G4 for the RSV-A dataset and TN + F + G4 for the RSV-B dataset, respectively [23].

## Results

### RSV seasonality pattern from 2010 to 2019

**Fig 1:**
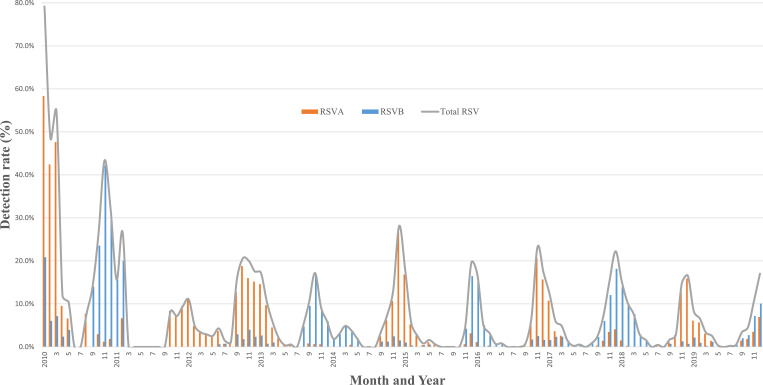
Seasonal outbreaks patterns and detection rate of RSV in Seoul from 2010 to 2019. Seasonal outbreaks patterns and detection rate of RSV in patients who visited Korea University Guro Hospital between January 2010 and December 2019. The detection rate of RSV-A and RSV-B was calculated as the percentage of positive cases detected among the total number of tests performed using either the multiplex RT-PCR (Seeplex® RV15 One-Step ACE Detection assay or Anyplex™ II 16 detection kit) or culture method. The x-axis of the graph represents the months of the year from January to December, while the y-axis represents the detection rate of RSV as a percentage. The orange line represents the detection rate of RSV-A, and the blue line represents the detection rate of RSV-B.

Distinct seasonality patterns were consistently observed over the 10-year study period from 2010 to 2019, with onset typically occurring in September during late fall or early winter and seasonal offset occurring in late spring around May in the Northern hemisphere. The peak in RSV activity was consistently observed between October and December across the study years (Fig 1). The highest detection rate for RSV-A occurred in January 2010, reaching 58.3%, while for RSV-B, the highest detection rate of 42.2% was observed in November 2010. However, the apparent increased detection rates in 2010 were due to the relatively smaller number of tests conducted compared to other years. Except for 2010, the highest detection rates for RSV-A and RSV-B were 15.9% to 26.6% and 10.1% to 20.0%, respectively. RSV-A and RSV-B dominated alternately, except in 2012; RSV-A was dominant in 2010, 2012, 2013, 2015, 2017, and 2019 epidemics, whereas RSV-B was dominant in 2011, 2014, 2016, and 2018 epidemics. The increased prevalence of RSV-A, starting in late 2011, was carried over in 2012 and 2013.

### Phylogenetic analysis

**Fig 2:**
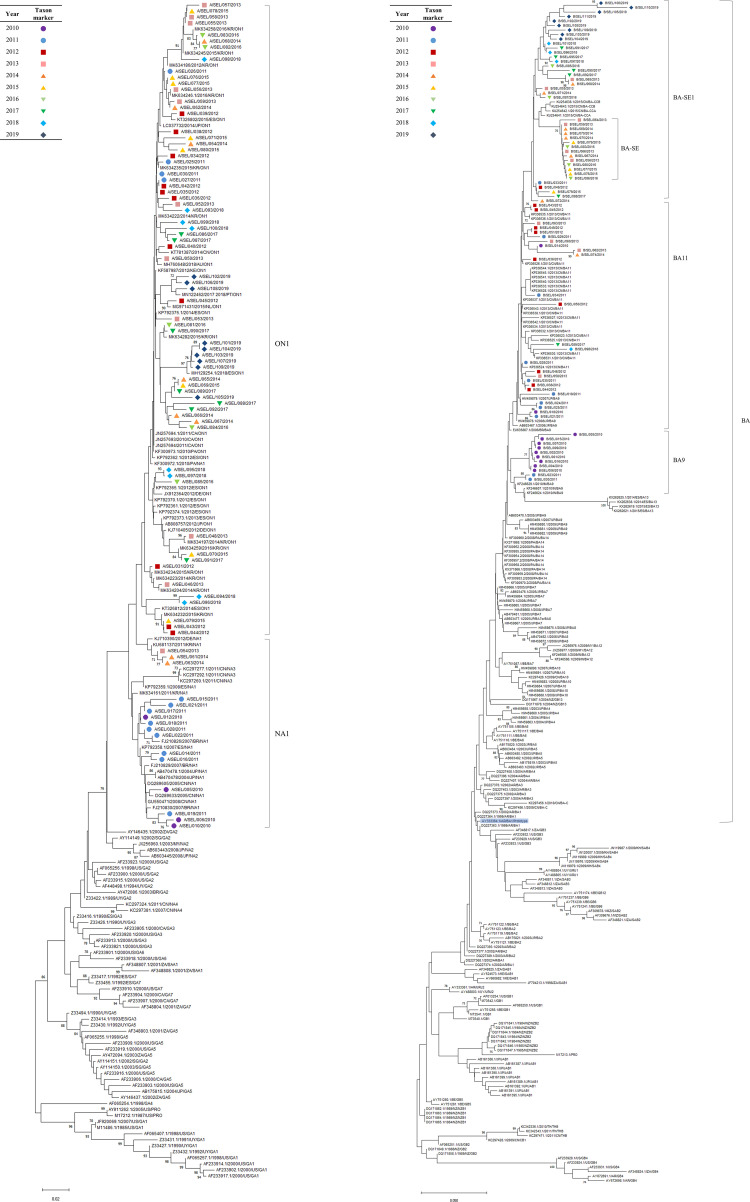
Neighbor-joining (NJ) phylogenetic tree of Seoul RSV-A and RSV-B strains. Phylogenetic trees based on the analysis of partial G gene sequence of RSV-positive samples from 2010–2019 in Seoul, (A) RSV-A and (B) RSV-B. Since the calculated average pairwise Jukes-Cantor distance was 0.08 and 0.06 for RSV-A and RSV-B, respectively, the data was suitable for building the NJ tree. The NJ trees were created using maximum composition likelihood and 1,000 bootstrap replicates by Mega X. Only bootstrap values ≥70% are shown for each tree. Strains are labeled with taxon markers of different colors and shapes depending on the collected year, as described in the table on the left-upper side of each NJ tree. Reference sequences are expressed in the following format; GenBank accession number /year/country/genotype. The prototype reference sequence for BA genotype (AY33364.1) was shaded in blue (B).

**Fig 3:**
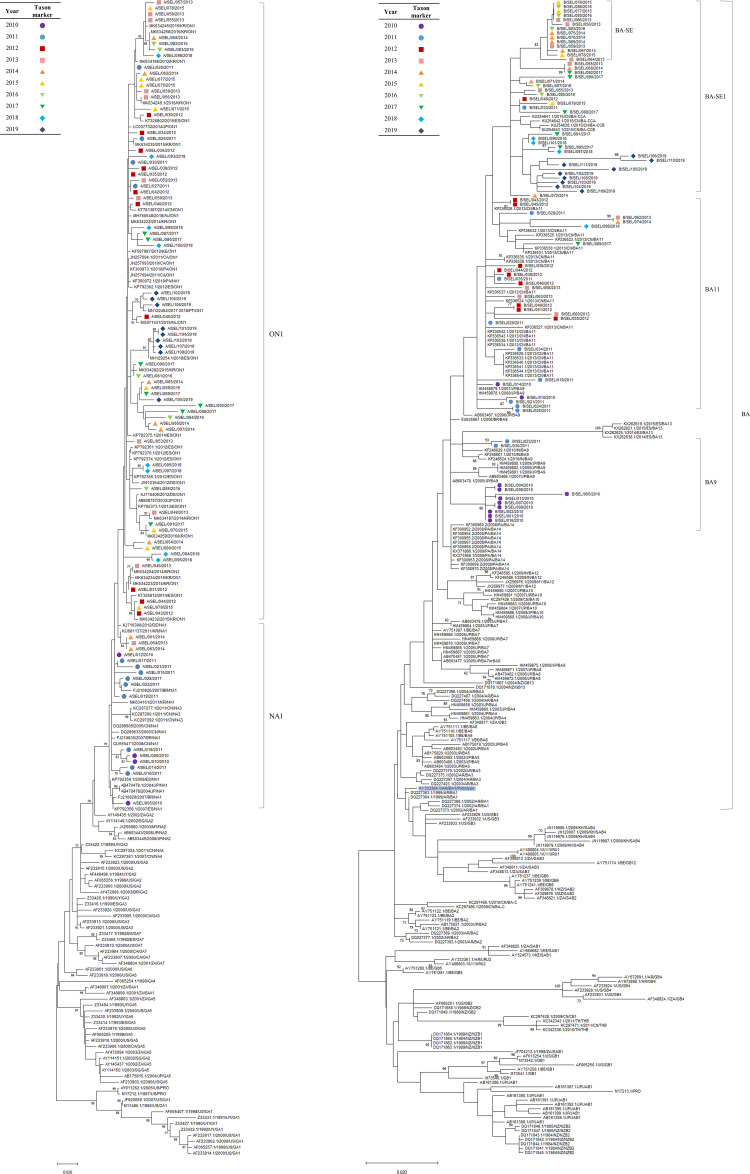
Maximum likelihood (ML) phylogenetic tree of Seoul RSV-A and RSV-B strains. ML phylogenetic trees based on the analysis of partial G gene sequence of RSV-positive samples from 2010–2019 in Seoul, (A) RSV-A and (B) RSV-B. The ML trees were created using the most suitable nucleotide substitution model (TN93 + G) with 1,000 bootstrap replicates by Mega X. Only bootstrap values ≥70% are shown for each tree. Strains are labeled with taxon markers of different colors and shapes depending on the collected year, as described in the table on the left-upper side of each ML tree. Reference sequences are expressed in the following format; GenBank accession number/year/country/genotype. The prototype reference sequence for BA genotype (AY33364.1) was shaded in blue (B).

**Fig 4:**
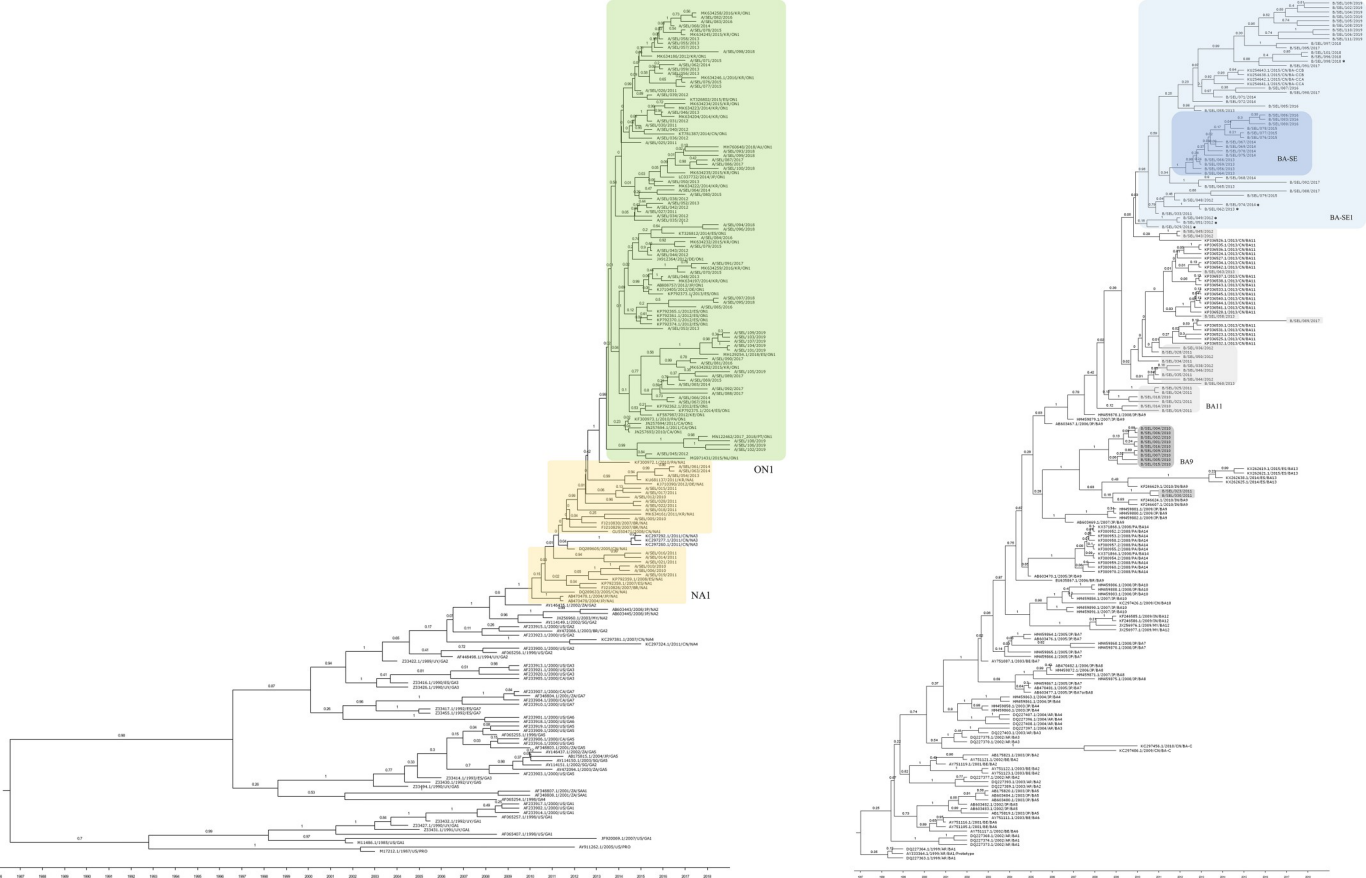
Maximum clade credibility (MCC) phylogenetic tree from Bayesian analysis of Seoul RSV-A and RSV-B strains. Time-scaled MCC trees based on the analysis of partial G gene sequence of RSV-positive samples from 2010–2019 in Seoul, (A) RSV-A and (B) RSV-B BA. (A) The RSV-A ON1 and NA1 genotypes were shaded in green and yellow boxes, respectively. The clusters of BA genotypes were shaded in mid-grey, light-grey, light-blue, and deep-blue boxes in BA9, BA11, BA-SE1, and BA-SE, respectively. The sequences showing discrepancies (BA-DIS) compared with the NJ and ML trees were marked with black asterisks in BA-SE1.

The partial G gene sequences of 84 RSV-A and 78 RSV-B samples were successfully obtained. All phylogenetic trees revealed two major groups for RSV-A and multiple groups for RSV-B (Figs 2–4). Seoul RSV-A sequences were classified into either ON1 (68/84, 80.9%) or NA1 (16/84, 19.0%) genotypes (Figs 2A–4A). The majority of sequences in NA1 (13/16, 81.3%) were collected in 2010 and 2011, representing a relatively earlier time point in the sample collection period.

On the other hand, Seoul RSV-B sequences showed diversified clusters within the BA genotype compared to Seoul RSV-A (Figs 2B–4B). First, most sequences in 2010 and two sequences in 2011 corresponded to the BA9 reference sequences (11/78, 14.1%). Secondly, sequences from various years were genetically close to the multiple BA11 reference sequences (26/78, 33.3%). Other than sequences showing association with BA9 or BA11, the remaining sequences were further classified into two lineages. The smaller lineage (14/78, 17.9%) close to BA-CCA/CCB subtypes formed a well-supported cluster with a bootstrap value of ≥70% in the NJ tree (Fig 2B). This cluster formed by these sequences, which we have designated as BA-SE (SE, for Seoul) for further analysis, was also supported by a bootstrap value of ≥70% in the ML tree and posterior probability ≥0.8 in the MCC tree, indicating a high degree of statistical support (Figs 3B and 4B). The remaining sequences (27/78, 34.6%) did not cluster clearly with any other specific BA genotypes and named BA-SE1. The clustering in the Bayesian MCC tree is consistent with the NJ and ML trees, except for six sequences (B/SEL/029, 049, 051, 062, 074, and 098) that were initially assigned to the BA11 (Fig 4B). These six sequences showing discrepancy were assigned to the BA-SE in the Bayesian MCC tree and assigned to BA-DIS.

The representative Seoul RSV sequences obtained in our study were submitted to GenBank and assigned accession numbers OQ511342—OQ511405 for RSV-A ON1 and OQ511406—OQ511475 for RSV-B BA genotype, respectively.

For further analysis of RSV-B strains, the *p*-distances including intragenotype and intergenotype distance was calculated. The highest intragenotypic distance observed in the dataset was 0.0446 in GB13. The intergenotypic distance above this threshold value were considered as indicative of a distinct genotype. The *p*-distances between Seoul BA9 and BA11 sequences and their respective reference sequences (BA9 and BA11) were below the threshold values of 0.0274 and 0.0200, respectively. Based on these results, the Seoul BA9 and BA11 sequences were assigned to the BA9 and BA11 genotype.

The intragenotypic distance of the BA-SE and BA-SE1 were 0.0037 and 0.0280, respectively. The intergenotypic distance between BA-CCA/CCB and BA-SE/SE1 were 0.0243, 0.0279, and 0.0276, 0.0275, respectively, which did not exceed the threshold value (Table 1). Also, the intergenotypic distance between BA-SE and BA-SE1 was below the threshold value, which was 0.0364. However, the *p*-distances between BA9 and BA-SE1/SE exceeded the threshold limit, suggesting that they belong to different genotypes. The intergenotypic distance of BA-DIS was also below the threshold value of 0.0446 compared with BA-SE, BA-SE1, BA9, and BA11.

**Table 1:** Intergenotypic *p*-distances in the partial G gene calculated between Seoul RSV-B strains.

	BA-SE	BA-SE1	BA-DIS	BA11	BA9
**BA-SE**					
**BA-SE1**	0.0364				
**BA-DIS**	0.0441	0.0401			
**BA11**	0.0388	0.0337	0.0301		
**BA9**	**0.0548**	**0.0473**	0.0441	0.0287	
**BA-CCB**	0.0279	0.0275	0.0409	0.0295	0.0435
**BA-CCA**	0.0243	0.0276	0.0386	0.0262	0.0403

A distinct genotype was assigned to clusters with intergenotypic distances greater than the highest intragenotypic distance observed for GB13 (0.0446). The value >0.0446 was highlighed in bold letters. Since BA-CCA/CCB cluster was found to be adjacent to BA-SE in the neighbor-joining tree, they have been included in the table for comparison.

### Characteristics of the deduced amino acid sequence with Shannon entropy analysis

**Fig 5:**
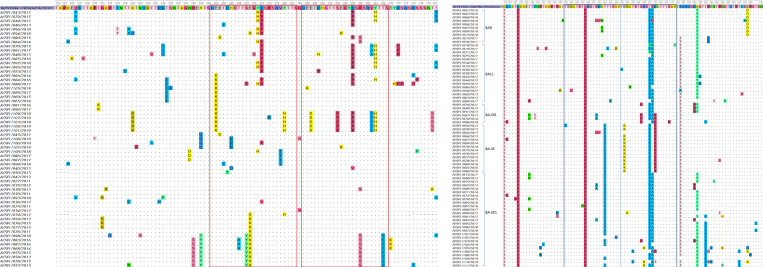
Deduced amino acid sequences in the 2^nd^ HVR of RSV G gene of Seoul RSV-A ON1 and RSV-B BA genotype. Amino acid sequences alignment of the partial G gene of RSV, (A) RSV-A ON1 and (B) RSV-B BA genotype. Sequences are expressed as RSV subtype (A or B)/city (Seoul, SEL)/sample number/collected year. Alignments are shown, and residues are numbered relative to sequences of (A) prototype ON1 strain ON67-1210A (GenBank accession no. JN257693) and (B) prototype strain BA strain BA4128/99B (GenBank accession no. AY333364). The two copies of 23 amino acid regions in the RSV-A group and 20 amino acid regions in the RSV-B group are framed in red and blue, respectively. Identical residues were indicated by dots. To enable convenient visualization of shared mutations, the RSV-A strains were arranged in the same order as depicted in the maximum likelihood (ML) tree. On the other hand, the RSV-B strains were listed according to their clusters in their trees (BA9, BA11, BA-DIS, BA-SE, and BA-SE1).

**Fig 6:**
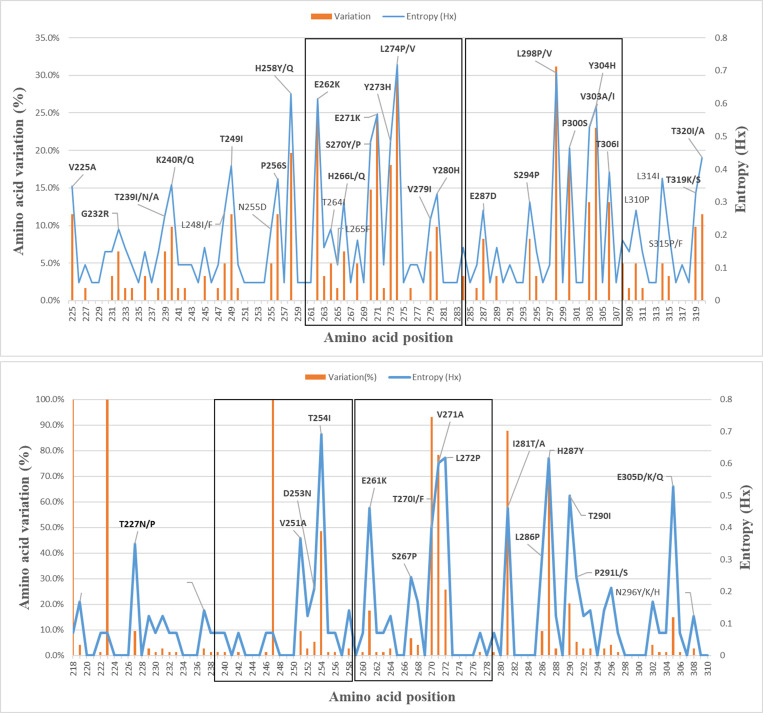
Shannon entropy plot combined with the frequency of amino acid variations of the 2^nd^ HVR of G gene in (A) RSV-A ON1 and (B) RSV-B BA genotype. Entropy values of each amino acid are calculated using BioEdit. Amino acid positions with the entropy value >0.2 are considered variable and denoted with a label. Among them, the amino acid positions with variation frequency > 5.0% and the entropy value 0.2 are highlighted in bold letters for easy reference. Duplicated regions in ON1 and BA are framed in black rectangles.

RSV-A ON1 has a characteristic 72 bp nt duplication in the 2^nd^ HVR of the G gene, which leads to duplication of 23 amino acids (QEETLHSTTSEGYLSPSQVYTTS, amino acid positions 261–283 and 285–307). All Seoul ON1 strains contained 23 duplicated amino acids (Fig 5A). Also, when listed according to their appearance in the ML tree, sequences located in adjacent clusters were found to share similar amino acid mutations. Among the observed variations, L298P (29.5%), L274P (27.9%), E262K (24.6%), E271K (24.6%), and Y304H (23.0%) were the most common (Table 2). These variations also showed high entropy values in the Shannon entropy plot ranging from 0.57 to 0.72, indicating their high variability (Fig 6A).

**Table 2:** Details of amino acid variations in the 2^nd^ HVR of RSV G gene of Seoul RSV-A ON1 and RSV-B BA genotype.

RSV subtype	Frequency	Amino acid variations
**RSV-A ON1**	>30%	L298P/V (31.1%)*
	>20–30%	E262K (24.6%), E271K (24.6%), L274P/V (29.5%)*, Y304H (23.0%)
	>10–20%	V225A (11.5%), T249I (11.5%), P256S (11.5%), P256S (11.5%), H258Y/Q (19.7%), S270Y/P (14.8%), Y273H (18.0%), P300S (18.0%), V303A/I (13.1%), T306I (13.1%), T320I/A (11.5%)
	>5–10%	G232R (6.6%), T239I/N/A (6.6%), K240R/Q (9.8%), H226L/Q (6.6%), V279I (6.6%), Y280H (9.8%), E287D (8.2%), S294P (8.2%), T319K/S (9.8%),
**RSV-B BA**	100%	K218T, L223P, S247P
	>50%	T270I/F (93.2%)*, V271A/G (78.4%)*, I281T/A (87.8%)*, H287Y (70.3%)
	>10–50%	T254I (50.0%), E261K (17.6%), L272P/T (25.7%), T290I (20.3%), E305D/K/Q (14.9%)
	>5–10%	T227N/P (9.5%), V251A/G (9.5%), D253N (5.4%), S267P (6.8%), L286P (9.5%), P291L/S (5.4%)

The amino acid variations detected at ≥ 5% frequency were arranged in increasing order of frequency and amino acid positions.

* L298P (29.5%)/L298V (1.6%), L274P (27.9%)/L2974V (1.6%), T270I (90.5%)/T270F (2.7%), V271A (77.0%)/T271G (1.4%), I281T (86.5%)/I281A (1.4%).

**Fig 7:**
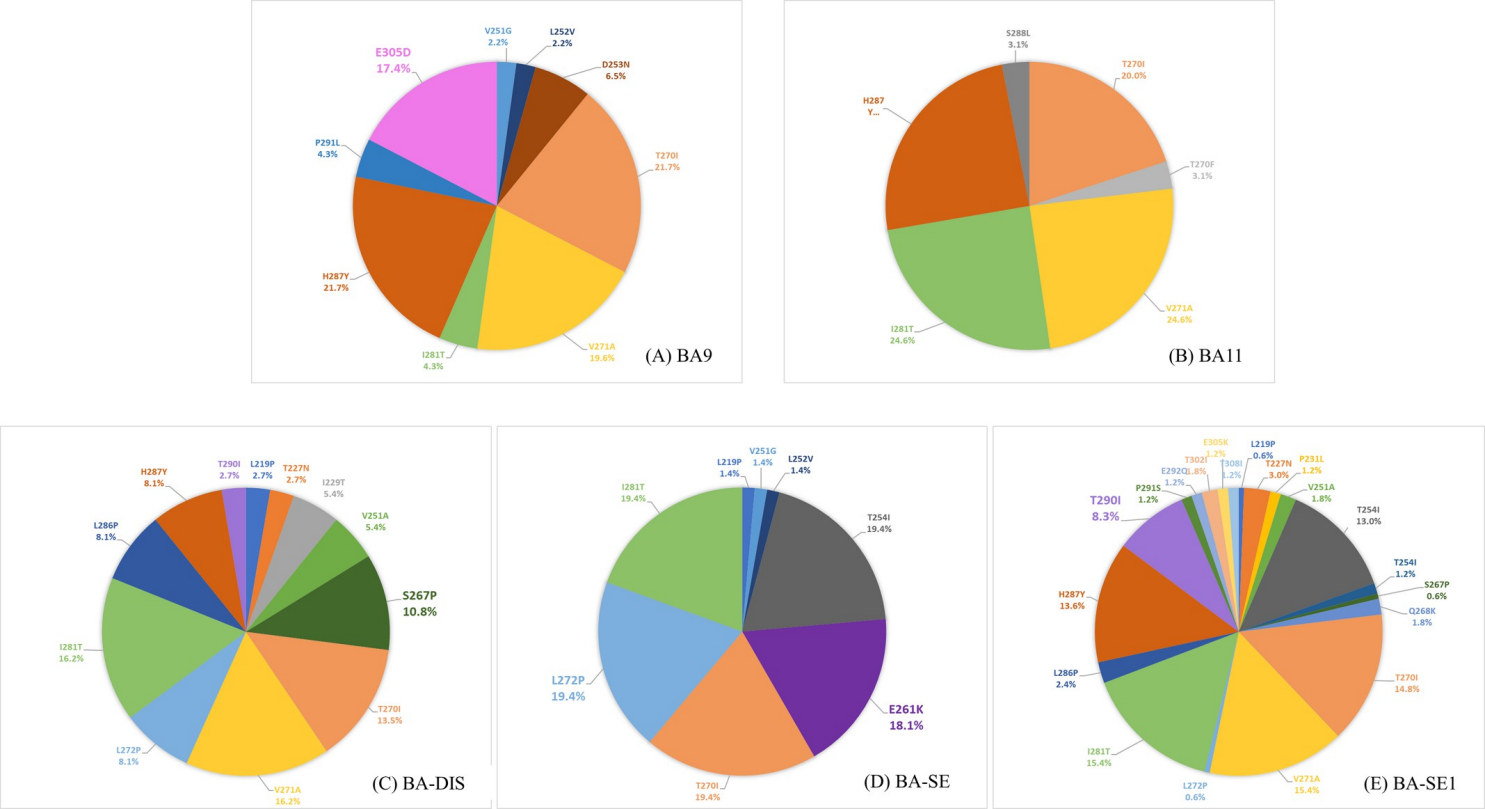
Proportion of amino acid variations in five Seoul RSV-B strains. The variations in amino acid sequences observed within the 2^nd^ HVR region of G gene of the Seoul RSV-B G gene were visually represented using different colors corresponding to the specific variations present, (A) BA9, (B) BA11, (C) BA-DIS, (D) BA-SE, and (E) BA-SE1. Unique variations that were not present (or hardly present) in other strains were highlighted in bold letters for each strain; (A) E305D in BA9, (C) S267P in BA-DIS, (D) E261K/L272P in BA-SE, and (E) T290I in BA-SE1.

RSV-B BA genotype has a 60 bp nt duplication in the 2^nd^ HVR of the G gene, which resulted in a duplication of 20 amino acid sequences (TERDTSTSQSTVLDTTTSKH). All stains in our study showed duplicated regions in the amino acid positions 240–259 and 260–279 (Fig 5B). K218T, L223P, S247P, and T270I mutations were observed in most Seoul RSV-B strains (90.5–100%). The substitutions reported as BA9 genotype-specific [32–34], V271A (77.0%), I281T (86.5%), and H287Y (70.3%), were observed with high entropy values ranging from 0.46 to 0.62 (Table 2 and Fig 6B). Distinct from the other four BA Seoul strains, BA-SE exhibited a distinct pattern of co-occurring variations, E261K and L272P, that were not observed in any other strains (Fig 5B). To facilitate the comparison of amino acid mutations among the five Seoul BA strains, the proportion of variations for each strain is visualized in Fig 7. Unique variations that were not present (or hardly present) in other strains were E305D in BA9, S267P in BA-DIS, E261K/L272P in BA-SE, and T290I in BA-SE1. BA-SE1 exhibited the highest number and complexity of amino acid variation types, while relatively few variation types were observed in BA11.

### Selection pressure analysis

Among the substitutions in ON1, substitutions at Y273H and L298P/V were predicted to be positively selected under three methods (Table 3). Site L274P/V was also under positive selection by SLAC and FUBAR analysis. The corresponding entropy values of Y273H, L298P/V, and L274P/V were 0.48, 0.69, and 0.72, respectively, which were all above the threshold limit of 0.2. The overall *d*N/*d*S ratio of ON1 strains estimated by SLAC was 0.85. On the other hand, no significant positive selection sites predicted by more than three methods were observed, accompanied by more negative selection sites in RSV-B BA strains. The site N296Y/K/H displayed positive selection by both the MEME and FUBAR methods, with an associated entropy value of 0.2119. The overall *d*N/*d*S ratio of BA was calculated as 0.47, which was lower than ON1.

**Table 3:** Positive and negative selection sites in the partial G gene of RSV-A ON1 and RSV-B BA genotype using SLAC, FEL and MEME with a p-value threshold of 0.05 and FUBAR with a posterior probability of 0.9.

RSV subtype	Methods	Positive selection sites	Negative selection sites
RSV-A ON1	SLAC	254, 274	none
	FEL	**273**, **298**	277, 283
	MEME	**273, 298**	n/a
	FUBAR	262, **273**, 274, **298**, 320	230, 277, 280, 283
RSV-B BA	SLAC	none	285
	FEL	none	226, 229, 234, 241, 252, 262, 278, 295, 307, 311
	MEME	296	n/a
	FUBAR	296	226, 229, 240, 238, 241, 252, 250, 262, 295, 307, 311

Positive selection sites by more than three methods are indicated in bold letters.

*SLAC: Single Likelihood ancestor Counting, FEL: Fixed-Effects Likelihood, MEME: Mixed Effects Model of Evolution, FUBAR: Fast, Unconstrained Bayesian AppRoximation. Positive selection sites by more than three methods are indicated in bold letters.

### Virus effective population analysis of the G gene in Korean RSV strains

The evolutionary rates of Korean RSV-A strains (n = 209) from 1999 to 2019 were calculated as 3.51 × 10^−3^ nt substitutions/site/year (95% HPD; 2.64–4.36 × 10^−3^). Among then, the evolutionary rates of ON1 was 1.42 × 10^−3^ nt substitutions/site/year (95% HPD; 0.89–1.98 × 10^−3^). The evolutionary rate of Korean RSV-B strains (n = 145) from 1991 and 2019 was 3.32 × 10^−3^ nt substitutions/site/year (95% HPD; 2.53–4.16× 10^−3^).

**Fig 8:**
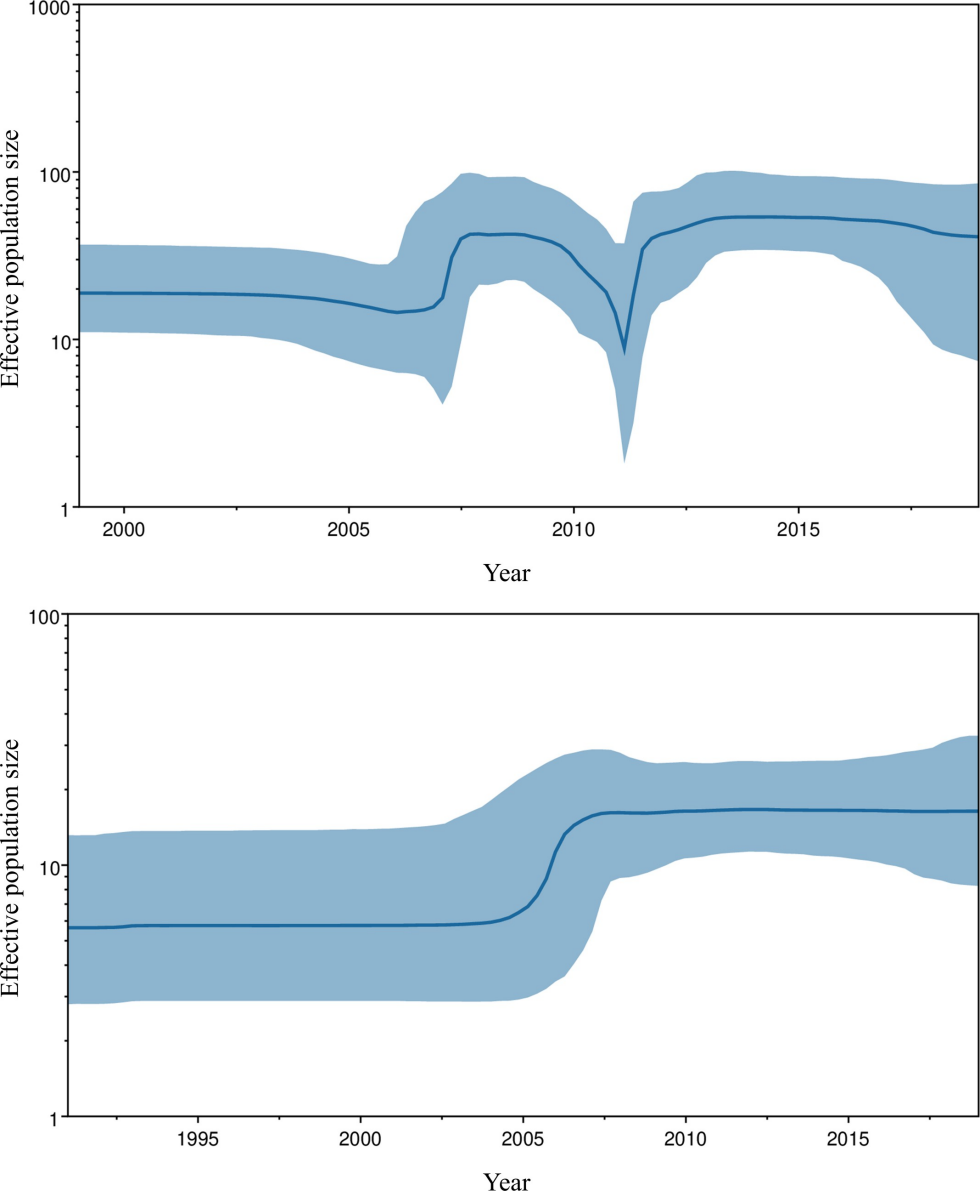
Bayesian skyline plot analysis revealing the temporal changes in effective population size of respiratory syncytial virus (RSV). Population dynamics of the G gene for (A) RSV-A and (B) RSV-B using Bayesian skyline plot analyses. The y-axis and x-axis represent the effective population size and the time in years, respectively. The estimated mean of the effective population size is represented by the bold line, while the colored margin indicates the 95% highest posterior density (HPD) interval.

BSP revealing the relative genetic diversity based on the effective population size (EPS) of RSV-A and RSV-B is presented in Fig 8. The EPS of RSV-A was relatively stable until 2006, but it rapidly increased between 2007 and 2008 (Fig 8A). This was followed by a sharp decrease starting in 2011, after which the EPS increased again and showed a steady trend until 2019. The first reported ON1 strain in Korea was isolated in August 2011 [35], followed by a second isolate in November of the same year, which became the dominant strain in 2012–2013 [15, 16]. Therefore, the emergence of the ON1 in 2011, along with the replacement of the NA1, is consistent with the observed fluctuation (S1 Fig). The EPS decreased slightly after the ON1 completely replaced the NA1. The EPS pattern in our study, characterized by an increase around 2006 and a more dramatic increase 2011, is consistent with previous analyses conducted in Korea [15]. The fluctuation observed between 2011 and 2013 is also consistent with a study conducted in Taiwan, which showed a similar pattern and coincided with the replacement of the NA1 genotype [36].

Meanwhile, the EPS of RSV-B remained relatively stable until 2005, followed by a gradual increase between 2005 and 2007, and then remained nearly constant until the end of the investigation period (Fig 8B). The BA9 genotype was isolated in the 2005/2006 season both in Japan and South Korea and subsequently became the predominant genotype by 2009 [8, 33]. The gradual increase in EPS observed between 2005 and 2007 coincided with the emergence of the BA9 genotype, similar to the changes observed in the ON1 genotype.

## Discussion

The G protein of RSV is one of the main antigens inducing a neutralizing immune response that reveals the highest degree of diversity and substitution rates in both subgroups [7]. Genetic and antigenic changes through positive selection in the C-terminal portion of the 2^nd^ HVR of the G gene of RSV could impact viral infectivity, survival, and ability to evade the host immune response [37]. In addition, it has been reported that specific genotypes of RSV are associated with increased disease severity [38, 39]. Therefore, we have aimed to analyze the molecular characterization of the RSV G gene focused on the 2^nd^ HVR for 10 consecutive years.

The epidemiology in Seoul showed distinct seasonality with onset in fall, a peak in late fall to December, and a season offset from May to June. This shifting pattern of dominance from one serotype to another in intervals of 1–3 years agrees with previous studies [34, 40–42]. The cyclical change in RSV subgroups is likely due to the acquired group immunity against the prevalent RSV subgroups in the previous year [43, 44]. Except for overestimation due to low number of tests in 2010, the average detection rate of RSV-A and RSV-B were 5.6% and 4.2%, respectively. This was similar to the previous study conducted between 2009 and 2014 in Korea [8]. The RSV-A detection rate peaked at 26.6% during the 2014/2015 season and the higher prevalence of RSV-A than RSV-B during 2011/2012 and 2012/2013 are all consistent with previous reports from Korea [16].

As reported in various countries, most of the Seoul RSV-A strains belonged to the ON1 (80.9%), and some of the Seoul RSV-B strains (14.1%) were clustered in the BA9 [44–49]. The duplication of the G gene observed in ON1 and BA provides the virus with benefits through increased cell attachment capacity [50, 51]. Although Seoul RSV-B sequences other than BA9 belonged to the BA genotype and were distinguishable from the non-BA genotype, they did not show an apparent clustering with any specific BA genotypes. The BA-SE sequences formed a distinct cluster with high statistical support (a bootstrap value of ≥70% and a posterior probability of ≥0.8) and intragenotypic *p*-distance of 0.0037, suggesting the potential emergence of a novel genotype. Previously, a new genotype of RSV has been identified when a set of sequences demonstrate a clustering pattern with a bootstrap value ≥70% and an intra-group p-distance <0.07, with a primary focus on the 2^nd^ HVR of G gene [1, 52–57]. Despite the general use of the G gene for genotype assignment, lack of consensus on the criteria used to define new genotypes across different studies further emphasized the need to discuss genotype assignments. Recent studies have suggested the necessity of a robust system that utilizes whole-genome sequencing (WGS) for future RSV genotyping [20, 58].

There are reports discussing more reliable consensus to be used to assign genotypes of RSV [10, 11, 19, 58]. Since our study utilized the partial G gene, the consensus methods proposed by Goya et al. and Muñoz-Escalante et al., which both utilize the G gene ectodomain, could be particularly relevant for assigning genotypes to Seoul RSV-B strains. Based on the criteria proposed by Goya et al., a new genotype can be defined as a monophyletic cluster of sequences with high statistical support, ≥80% bootstrap value and ≥0.8 posterior probability with the intergenotypic *p*-distance ≥0.05 for RSV-B [19]. The intergenotypic *p*-distance of Seoul RSV-B including BA-SE, BA-SE1, and BA-DIS did not exceeded the threshold value of 0.05. Muñoz-Escalante et al. proposed a different approach for RSV genotype classification, which involves using the highest intragenotypic *p*-distance as the minimum threshold to define a genotype [10, 11]. The highest intragenetypic *p*-distance was 0.0446 in GB13 in our dataset. All the intergenotypic *p*-distance of Seoul RSV-A strains were below the threshold value of 0.0446, except for between BA-SE/SE1 and BA9. While BA-SE met the criteria for new genotype assignment under the previous classification, there is currently insufficient evidence to support the assignment of a new genotype using more recent classification methods. Based on the intergenotypic *p*-distance between BA-SE/SE1 and BA9 exceeding the threshold value, it is unlikely that BA-SE/SE1 belongs to the BA9 genotype. Moreover, their position in phylogenetic trees suggests that assigning them to the BA11 or BA-CC genotype would be more appropriate, given their intergenotypic *p*-distances below the threshold value. According to a recent global study, the BA-CC genotype has emerged as the dominant RSV-B genotype, surpassing the previously leading BA genotype [59].

In the NJ tree that incorporated all reference sequences, including Korean RSV sequences, the Seoul RSV-A strains tended to cluster together with other Korea strains than with strains from other countries (S2 Fig). This finding is consistent with the previous study, which suggested that the evolution of Korea ON1 occurred independently in the domestic niche following its initial introduction. In contrast, the analysis of Seoul RSV-B strains showed a low level of closeness to Korean strains, and the BA-SE sequences did not form clusters with any other reference sequences (S3 Fig). Further analysis of the RSV genome through WGS of other genes using a consensus classification system could help to determine if the BA-SE represents a novel genotype.

In the level of amino acids, the change in the average number of variations per year was remarkable in the RSV-A ON1 compared to that of the RSV-B BA (S4 Fig). The variations in the ON1 fluctuated year by year with on the rise in the long term but generally maintained less average number than that of the BA. But ON1 strains were more diverse than BA at the amino acid level according to the *d*N/*d*S ratio and selective pressure analysis, suggesting that the G gene of ON1 is under immune pressure selection [60]. Compared to the ON1 genotype, the Seoul RSV-B BA strains exhibited a relatively low *d*N/*d*S ratio with one positive selection site, amino acid 296, which had been previously reported [61]. Although there were some unique amino acid mutations in the 2nd HVR of Seoul RSV-B, revealing their variability, most substitutions are likely neutral. The negative selection sites were observed more in BA than ON1. The presence of these negative selection sites may have a role in the prevention of deterioration of protein and antigenic function [62].

When integrating the mutated sites with a Shannon entropy value of >0.2 and positive selection by at least two methods, three substitution sites in ON1 (273, 274, and 298) significantly reflected their variability, which suggests frequent substitutions occurred at their position. Among them, L274P mutation has been reported to be associated with viral immune escape [13]. Y273H and L298P substitutions were reported to be under positive selection pressure in northeastern China [63] and Taiwan ON1 strains [36], respectively. Since the positive or negative selection sites may differ depending on the selection method or studied strains from study to study, these commonly observed positive selection sites are considered meaningful.

The mean evolutionary rates estimated for the G gene of Korean RSV-A strains from 1999 to 2019 were 3.51 × 10^−3^ nt substitutions/site/year, which was within the reported range in previous studies, 1.48–4.68 × 10^−3^ nt substitutions/site/year [64–66]. The evolutionary rate of the ON1 genotype (1.42 × 10^−3^ nt substitutions/site/year) was lower than those of the previously reported mean evolutionary rate of the global ON1 (4.10 × 10^−3^ nt substitutions/site/year) [67]. The evolutionary rate can vary depending on the characteristics in each region, like population density, and is affected by a limited host range and immune variations [67]. Therefore, this may be explained by the characteristics of the strains collected within the region restricted to Seoul andis consistent with the clustering of Korean RSV-A strains in the NJ tree, indicating their tendency to group together rather than with other foreign strains. Meanwhile, the evolutionary rate for Korean RSV-B was 3.32 × 10^−3^ nt substitutions/site/year, which was in the range of previously reported estimates in BA genotype, 1.92 × 10^−3^ to 5.89 × 10^−3^ nt substitutions/site/year [64–66].

In the BSP of Korean RSV-A, the abrupt first increase curve observed in 2007–2008 occurred at a similar timeline with the increased prevalence of the NA1, and the second increase curve coincided with the emergence and prevalence of the ON1. The abrupt fluctuation between 2011 and 2012 due to the switch of the prevalent genotype from NA1 to ON1 was also observed in other studies [36, 68]. On the other hand, the population dynamics of Korean RSV-B were relatively stable except for the slow increase from 2005 to 2007. The genotypes present before appeared to have been replaced by BA9 since then [1]. These results suggest that population dynamics tend to be affected by the emergence of the new RSV genotypes.

This study has some limitations. Firstly, our analysis was restricted to the 2nd HVR of the G gene, potentially resulting in the exclusion of important information from other regions. Consequently, the conclusion regarding the genotype assignment of the BA-SE remains inconclusive. Limited data from a limited cohort may be one of the restrictions of local studies. Systematic studies of large datasets can provide a broad perspective, enabling researchers to see the bigger picture rather than focusing solely on individual components. Secondly, a few samples were excluded due to either unsuccessful sequencing or inadequate length to analyze the 2nd HVR region. This may have resulted from the low viral load of the samples due to prolonged storage in a refrigerated state. Finally, the comparison of disease severity based on specific genotypes could not be achieved due to the unavailability of patient data. These limitations indicate the need for further research, including a systematic approach to address these gaps and provide a more comprehensive understanding.

Despite the limitation of our study, it is crucial to maintain surveillance by monitoring occurrence from a local perspective to facilitate further study. The coronavirus disease 2019 pandemic was followed by unexpected outbreaks of RSV with different seasonal patterns, as reported by previous studies [69–72]. By monitoring these patterns locally, we can identify potential outbreaks, comprehend transmission dynamics, and develop effective strategies to prevent future epidemics.

## Conclusion

In conclusion, RSV-A ON1 and RSV-B BA genotypes are the dominant genotypes in Seoul between 2010 and 2019. Some unique characteristics of Seoul RSV strains were observed, including a relatively low evolutionary rate of RSV-A ON1 and RSV-B sequences that did not cluster with other BA genotypes in the phylogenetic trees. Further WGS analyses using other RSV genes and a consensus classification criteria may be required to assign the BA-SE as a novel genotype. Continuous surveillance of the molecular epidemiology of local RSV strains is necessary to track epidemic patterns and prepare for the emergence of new strains. The findings from this study are expected to contribute to our understanding of the dynamic process of RSV evolution and provide insight into the genetic diversity and prevalence of RSV strains in Korea.

## Supporting information

S1 Table GenBank accession number, country, and genotype of RSV-A reference sequences. (DOCX)

S2 Table GenBank accession number, country, and genotype of RSV-B reference sequences. (DOCX)

S1 Fig Bayesian skyline plot (BSP) of Korean RSV-A with maximum clade credibility (MCC) tree. Population dynamics of the G gene for Korean RSV-A, depicted in the down panel of the MCC tree (upper panel), using the same time scale as the Bayesian skyline plot (BSP). Notably, the fluctuated pattern observed between 2011 and 2013 coincides with the emergence of the RSV-ON1 genotype.
(TIF)

S2 Fig Neighbor-joining (NJ) phylogenetic tree of Seoul RSV-A strains incorporating all reference sequences. (TIF)

S3 Fig Neighbor-joining (NJ) phylogenetic tree of Seoul RSV-B strains incorporating all reference sequences. The NJ phylogenetic trees using the partial G gene sequence of Seoul RSV-A (S2 Fig) and RSV-B (S3 Fig), with their corresponding reference sequences. The NJ trees were created using maximum composition likelihood, and 1,000 bootstrap replicates by Mega X. Only bootstrap values ≥ 70% are shown for each tree. Strains are labeled with taxon markers of different colors and shapes depending on the collected year, as described in the table on the left-upper side of the tree. The Korean reference sequences (n = 67) were marked in blank black circles.
(TIF)

S4 Fig Change of an average number of variations in RSV-A ON1 and RSV-B BA genotypes by year. The number of variations in each sequence of Seoul RSV strains was determined compared to the reference sequences, prototype ON1 strain ON67-1210A and BA strain BA4128/99B, respectively.
(TIF)
