# Supplementary material for: Molecular characterization of human respiratory syncytial virus in Seoul, South Korea, during 10 consecutive years, 2010–2019
Source: PLoS One. 2023 Apr 6;18(4):e0283873. doi: 10.1371/journal.pone.0283873 (PMC10079039; doi:10.1371/journal.pone.0283873)
Supplement: S1 Table — (DOCX) [file pone.0283873.s001.docx]

**S Table 1.**

| GenBank accession number | Country | Genotype |
| --- | --- | --- |
| AF065257.1 | USA | GA1 |
| AF065407.1 | USA | GA1 |
| AF233902.1 | USA | GA1 |
| AF233914.1 | USA | GA1 |
| AF233917.1 | USA | GA1 |
| JF920069.1 | USA | GA1 |
| M11486.1 | USA | GA1 |
| Z33427.1 | Uruguay | GA1 |
| Z33431.1 | Uruguay | GA1 |
| Z33432.1 | Uruguay | GA1 |
| AF065256.1 | USA | GA2 |
| AF233900.1 | USA | GA2 |
| AF233915.1 | USA | GA2 |
| AF233923.1 | USA | GA2 |
| AF448498.1 | Uruguay | GA2 |
| AY114149.1 | Singapore | GA2 |
| AY114151.1 | Singapore | GA2 |
| AY146435.1 | South Africa | GA2 |
| AY472086.1 | Brazil | GA2 |
| Z33422.1 | Uruguay | GA2 |
| AF233905.1 | Canada | GA3 |
| AF233913.1 | USA | GA3 |
| AF233920.1 | USA | GA3 |
| AF233921.1 | USA | GA3 |
| Z33414.1 | Spain | GA3 |
| Z33416.1 | Spain | GA3 |
| Z33426.1 | Uruguay | GA3 |
| AF065254.1 | USA | GA4 |
| AB175815.1 | Japan | GA5 |
| AF065255.1 | USA | GA5 |
| AF233903.1 | USA | GA5 |
| AF233906.1 | Canada | GA5 |
| AF233909.1 | USA | GA5 |
| AF233916.1 | USA | GA5 |
| AF233919.1 | USA | GA5 |
| AF348803.1 | South Africa | GA5 |
| AY114150.1 | Singapore | GA5 |
| AY146437.1 | South Africa | GA5 |
| AY472094.1 | South Africa | GA5 |
| Z33430.1 | Uruguay | GA5 |
| Z33494.1 | Uruguay | GA5 |
| AF233901.1 | USA | GA6 |
| AF233918.1 | USA | GA6 |
| AF233904.1 | Canada | GA7 |
| AF233907.1 | Canada | GA7 |
| AF233910.1 | USA | GA7 |
| AF348804.1 | South Africa | GA7 |
| Z33417.1 | Spain | GA7 |
| Z33455.1 | Spain | GA7 |
| AB470478.1 | Japan | NA1 |
| KF300972.1 | Panama | NA1 |
| KP792358.1 | Spain | NA1 |
| KP792359.1 | Spain | NA1 |
| AB470478 | Japan | NA1 |
| DQ289605 | China | NA1 |
| DQ289633 | China | NA1 |
| FJ210826 | Brazil | NA1 |
| FJ210829 | Brazil | NA1 |
| FJ210830 | Brazil | NA1 |
| GU550471 | China | NA1 |
| KJ710390 | Germany | NA1 |
| KU681137 | Korea | NA1 |
| MK634161 | Korea | NA1 |
| JX256960.1 | Malaysia | NA2 |
| AB603443 | Japan | NA2 |
| AB603445 | Japan | NA2 |
| KC297260.1 | China | NA3 |
| KC297277.1 | China | NA3 |
| KC297292.1 | China | NA3 |
| KC297324.1 | China | NA4 |
| KC297381.1 | China | NA4 |
| JN257694.1 | Canada | ON1 |
| KF300973.1 | Panama | ON1 |
| KP792361.1 | Spain | ON1 |
| KP792362.1 | Spain | ON1 |
| KP792365.1 | Spain | ON1 |
| KP792370.1 | Spain | ON1 |
| KP792373.1 | Spain | ON1 |
| KP792374.1 | Spain | ON1 |
| KP792375.1 | Spain | ON1 |
| AB808757 | Japan | ON1 |
| JN257693 | Canada | ON1 |
| JN257694 | Canada | ON1 |
| JX912364 | Germany | ON1 |
| KF587987 | Kenya | ON1 |
| KJ710405 | Germany | ON1 |
| KT326802 | Spain | ON1 |
| KT326812 | Spain | ON1 |
| KT781387 | China | ON1 |
| LC037732 | Japan | ON1 |
| MG971431 | Netherlands | ON1 |
| MH129254 | Spain | ON1 |
| MH760648 | Australia | ON1 |
| MK634186 | Korea | ON1 |
| MK634197 | Korea | ON1 |
| MK634204 | Korea | ON1 |
| MK634222 | Korea | ON1 |
| MK634223 | Korea | ON1 |
| MK634232 | Korea | ON1 |
| MK634234 | Korea | ON1 |
| MK634235 | Korea | ON1 |
| MK634245 | Korea | ON1 |
| MK634246.1 | Korea | ON1 |
| MK634258 | Korea | ON1 |
| MK634259 | Korea | ON1 |
| MK634282 | Korea | ON1 |
| MN122462 | Portugal | ON1 |
| AY911262.1 | USA | PRO |
| M17212.1 | USA | PRO |
| AF348807.1 | South Africa | SAA1 |
| AF348808.1 | South Africa | SAA1 |
