# Supplementary material for: Molecular characterization of human respiratory syncytial virus in Seoul, South Korea, during 10 consecutive years, 2010–2019
Source: PLoS One. 2023 Apr 6;18(4):e0283873. doi: 10.1371/journal.pone.0283873 (PMC10079039; doi:10.1371/journal.pone.0283873)
Supplement: S2 Table — (DOCX) [file pone.0283873.s002.docx]

**S Table 2.**

| GenBank accession number | Country | Genotype |
| --- | --- | --- |
| AY333364.1 | Argentina | BA1 |
| DQ227363.1 | Argentina | BA1 |
| DQ227364.1 | Argentina | BA1 |
| DQ227368.1 | Argentina | BA1 |
| DQ227373.1 | Argentina | BA1 |
| DQ227374.1 | Argentina | BA1 |
| HM459883.1 | Japan | BA10 |
| HM459884.1 | Japan | BA10 |
| HM459886.1 | Japan | BA10 |
| HM459888.1 | Japan | BA10 |
| HM459890.1 | Japan | BA10 |
| HM459891.1 | Japan | BA10 |
| KC297426.1 | China | BA10 |
| KP336523.1 | China | BA11 |
| KP336524.1 | China | BA11 |
| KP336525.1 | China | BA11 |
| KP336526.1 | China | BA11 |
| KP336527.1 | China | BA11 |
| KP336528.1 | China | BA11 |
| KP336530.1 | China | BA11 |
| KP336531.1 | China | BA11 |
| KP336532.1 | China | BA11 |
| KP336533.1 | China | BA11 |
| KP336534.1 | China | BA11 |
| KP336535.1 | China | BA11 |
| KP336536.1 | China | BA11 |
| KP336537.1 | China | BA11 |
| KP336538.1 | China | BA11 |
| KP336540.1 | China | BA11 |
| KP336541.1 | China | BA11 |
| KP336542.1 | China | BA11 |
| KP336543.1 | China | BA11 |
| KP336544.1 | China | BA11 |
| KP336545.1 | China | BA11 |
| JX256976.1 | Malaysia | BA12 |
| JX256977.1 | Malaysia | BA12 |
| KF246585.1 | India | BA12 |
| KF246586.1 | India | BA12 |
| KX262619.1 | Spain | BA13 |
| KX262621.1 | Spain | BA13 |
| KX262625.1 | Spain | BA13 |
| KX262638.1 | Spain | BA13 |
| KF300952.2 | Panama | BA14 |
| KF300953.2 | Panama | BA14 |
| KF300954.2 | Panama | BA14 |
| KF300955.2 | Panama | BA14 |
| KF300957.2 | Panama | BA14 |
| KF300958.2 | Panama | BA14 |
| KF300959.2 | Panama | BA14 |
| KF300960.2 | Panama | BA14 |
| KF300970.2 | Panama | BA14 |
| KX371866.1 | Panama | BA14 |
| KX371868.1 | Panama | BA14 |
| AB175821.1 | Japan | BA2 |
| AY751119.1 | Belgium | BA2 |
| AY751121.1 | Belgium | BA2 |
| AY751122.1 | Belgium | BA2 |
| AY751123.1 | Belgium | BA2 |
| DQ227377.1 | Argentina | BA2 |
| DQ227389.1 | Argentina | BA2 |
| DQ227393.1 | Argentina | BA2 |
| DQ227370.1 | Argentina | BA3 |
| DQ227375.1 | Argentina | BA3 |
| DQ227397.1 | Argentina | BA3 |
| DQ227403.1 | Argentina | BA3 |
| DQ227396.1 | Argentina | BA4 |
| DQ227407.1 | Argentina | BA4 |
| DQ227408.1 | Argentina | BA4 |
| HM459858.1 | Japan | BA4 |
| HM459860.1 | Japan | BA4 |
| HM459861.1 | Japan | BA4 |
| HM459863.1 | Japan | BA4 |
| AB175819.1 | Japan | BA5 |
| AB175820.1 | Japan | BA5 |
| AB603480.1 | Japan | BA5 |
| AB603482.1 | Japan | BA5 |
| AB603483.1 | Japan | BA5 |
| AB603484.1 | Japan | BA5 |
| AY751105.1 | Belgium | BA6 |
| AY751111.1 | Belgium | BA6 |
| AY751116.1 | Belgium | BA6 |
| AY751117.1 | Belgium | BA6 |
| AB470481.1 | Japan | BA7 |
| AB603476.1 | Japan | BA7 |
| AY751087.1 | Belgium | BA7 |
| HM459864.1 | Japan | BA7 |
| HM459865.1 | Japan | BA7 |
| HM459866.1 | Japan | BA7 |
| HM459867.1 | Japan | BA7 |
| HM459868.1 | Japan | BA7 |
| HM459870.1 | Japan | BA7 |
| AB470482.1 | Japan | BA8 |
| AB603477.1 | Japan | BA8 |
| HM459871.1 | Japan | BA8 |
| HM459872.1 | Japan | BA8 |
| HM459875.1 | Japan | BA8 |
| AB603467.1 | Japan | BA9 |
| AB603469.1 | Japan | BA9 |
| AB603470.1 | Japan | BA9 |
| EU635867.1 | Brazil | BA9 |
| HM459878.1 | Japan | BA9 |
| HM459880.1 | Japan | BA9 |
| HM459881.1 | Japan | BA9 |
| HM459882.1 | Japan | BA9 |
| KF246607.1 | India | BA9 |
| KF246624.1 | India | BA9 |
| KF246629.1 | India | BA9 |
| HM459879.1 | Japan | BA9 |
| KC297456.1 | China | BA-C |
| KC297486.1 | China | BA-C |
| KU254641.1 | China | BA-CCA |
| KU254642.1 | China | BA-CCA |
| KU254638.1 | China | BA-CCB |
| KU254643.1 | China | BA-CCB |
| KC297428.1 | China | CB1 |
| AF013254.1 | USA | GB1 |
| AF065250.1 | USA | GB1 |
| AY751256.1 | Belgium | GB1 |
| M73540.1 | USA | GB1 |
| M73541.1 | USA | GB1 |
| M73542.1 | USAA | GB1 |
| AY751174.1 | Belgium | GB12 |
| DQ171867.1 | New Zealand | GB13 |
| DQ171878.1 | New Zealand | GB13 |
| AF065251.1 | USA | GB2 |
| DQ171849.1 | New Zealand | GB2 |
| DQ171858.1 | New Zealand | GB2 |
| AF233929.1 | USA | GB3 |
| AF233932.1 | USA | GB3 |
| AF233933.1 | USA | GB3 |
| AF348817.1 | South Africa | GB3 |
| AF233924.1 | USA | GB4 |
| AF233928.1 | USA | GB4 |
| AF233931.1 | USA | GB4 |
| AF348824.1 | South Africa | GB4 |
| AY672691.1 | Argentina | GB4 |
| AY672698.1 | Argentina | GB4 |
| AY751280.1 | Belgium | GB5 |
| AY751281.1 | Belgium | GB5 |
| AY751237.1 | Belgium | GB6 |
| AY751239.1 | Belgium | GB6 |
| AY751241.1 | Belgium | GB6 |
| AB161386.1 | Japan | JAB1 |
| AB161387.1 | Japan | JAB1 |
| AB161388.1 | Japan | JAB1 |
| AB161389.1 | Japan | JAB1 |
| AB161390.1 | Japan | JAB1 |
| AB161391.1 | Japan | JAB1 |
| AB161392.1 | Japan | JAB1 |
| AB161395.1 | Japan | JAB1 |
| AB161399.1 | Japan | JAB1 |
| DQ171862.1 | New Zealand | NZB1 |
| DQ171863.1 | New Zealand | NZB1 |
| DQ171864.1 | New Zealand | NZB1 |
| DQ171865.1 | New Zealand | NZB1 |
| DQ171841.1 | New Zealand | NZB2 |
| DQ171842.1 | New Zealand | NZB2 |
| DQ171843.1 | New Zealand | NZB2 |
| DQ171844.1 | New Zealand | NZB2 |
| DQ171845.1 | New Zealand | NZB2 |
| DQ171846.1 | New Zealand | NZB2 |
| DQ171847.1 | New Zealand | NZB2 |
| M17213.1 | USA | PRO |
| AF348825.1 | South Africa | SAB1 |
| AY524573.1 | Kenya | SAB1 |
| AY660682.1 | Kenya | SAB1 |
| JF704213.1 | South Africa | SAB1 |
| AF309676.1 | Mozambique | SAB2 |
| AF309678.1 | Mozambique | SAB2 |
| AF348821.1 | South Africa | SAB2 |
| AF348811.1 | South Africa | SAB3 |
| AF348812.1 | South Africa | SAB3 |
| AF348813.1 | South Africa | SAB3 |
| JN119976.1 | Cambodia | SAB4 |
| JN119979.1 | Cambodia | SAB4 |
| JN119987.1 | Cambodia | SAB4 |
| JN119989.1 | Cambodia | SAB4 |
| JN120007.1 | Cambodia | SAB4 |
| KC297471.1 | China | THB |
| KC342336.1 | Thailand | THB |
| KC342343.1 | Thailand | THB |
| AY488804.1 | Uruguay | URU1 |
| AY488805.1 | Uruguay | URU1 |
| AY333361.1 | Argentina | URU2 |
| AY488803.1 | Uruguay | URU2 |
