# Supplementary figures and images for: Molecular characterization of human respiratory syncytial virus in Seoul, South Korea, during 10 consecutive years, 2010–2019
Source: PLoS One. 2023 Apr 6;18(4):e0283873. doi: 10.1371/journal.pone.0283873 (PMC10079039; doi:10.1371/journal.pone.0283873)

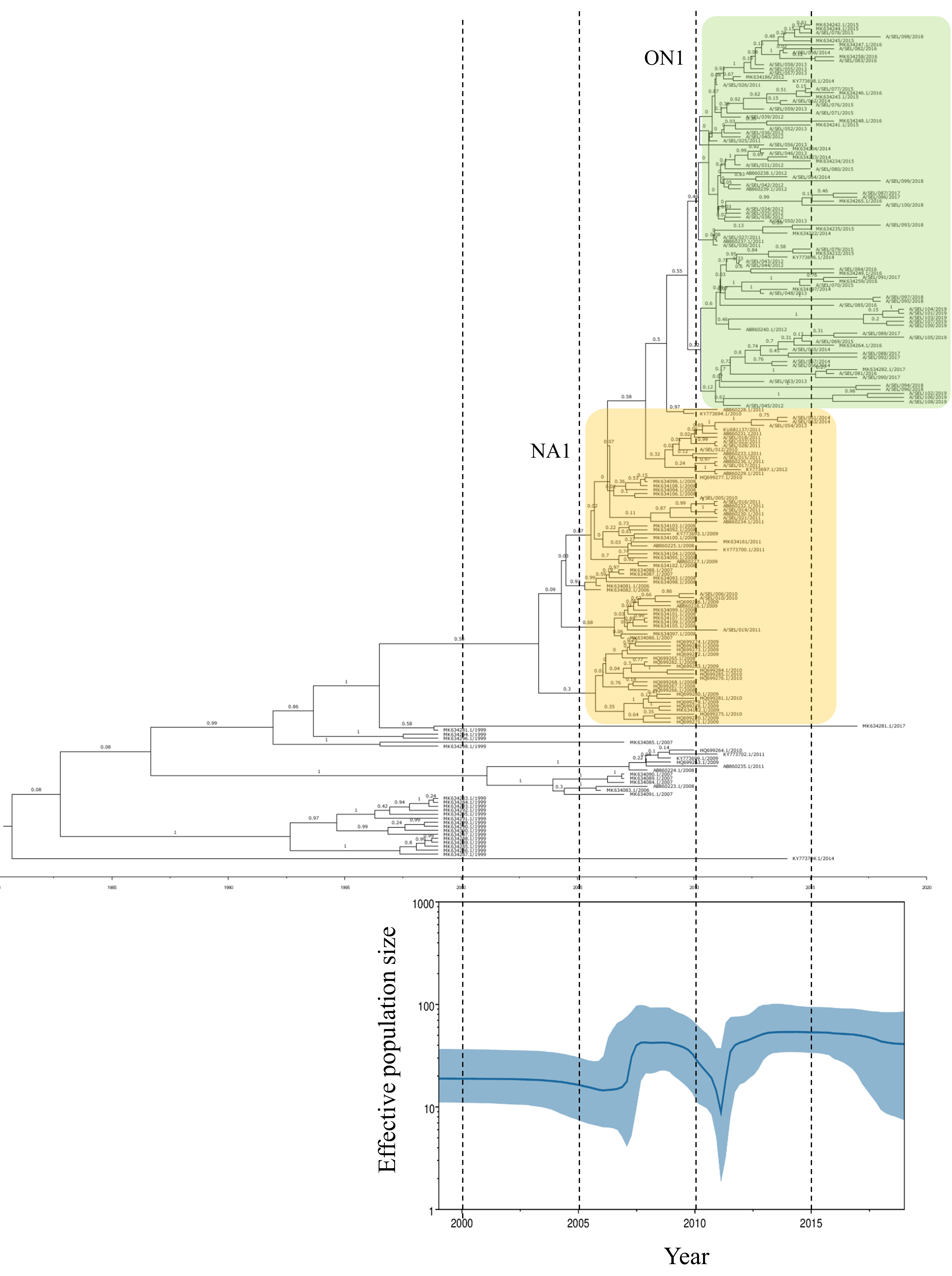

Supplement: S1 Fig — Population dynamics of the G gene for Korean RSV-A, depicted in the down panel of the MCC tree (upper panel), using the same time scale as the Bayesian skyline plot (BSP). Notably, the fluctuated pattern observed between 2011 and 2013 coincides with the emergence of the RSV-ON1 genotype. (TIF) [file pone.0283873.s003.tif]

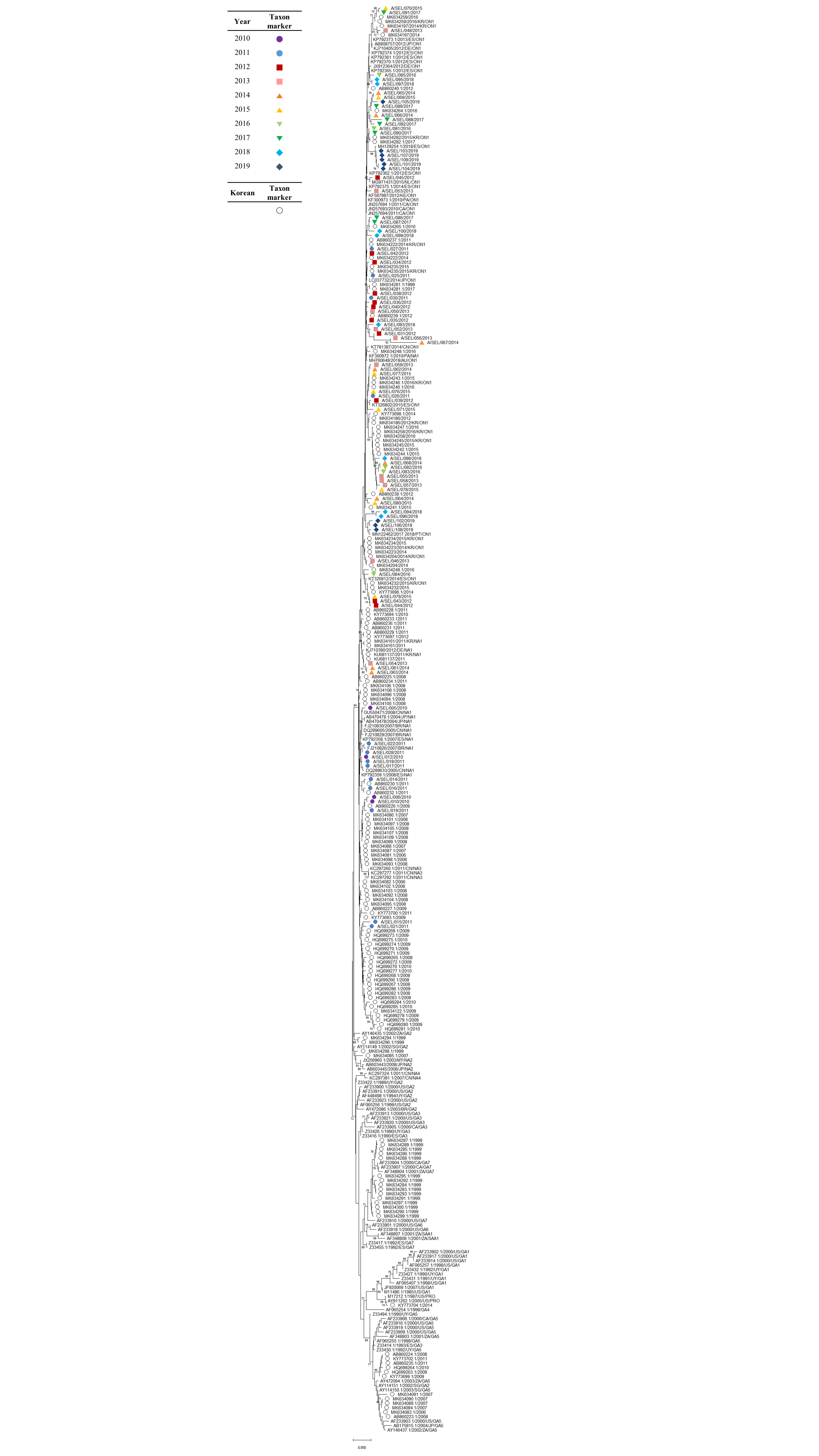

Supplement: S2 Fig — (TIF) [file pone.0283873.s004.tif]

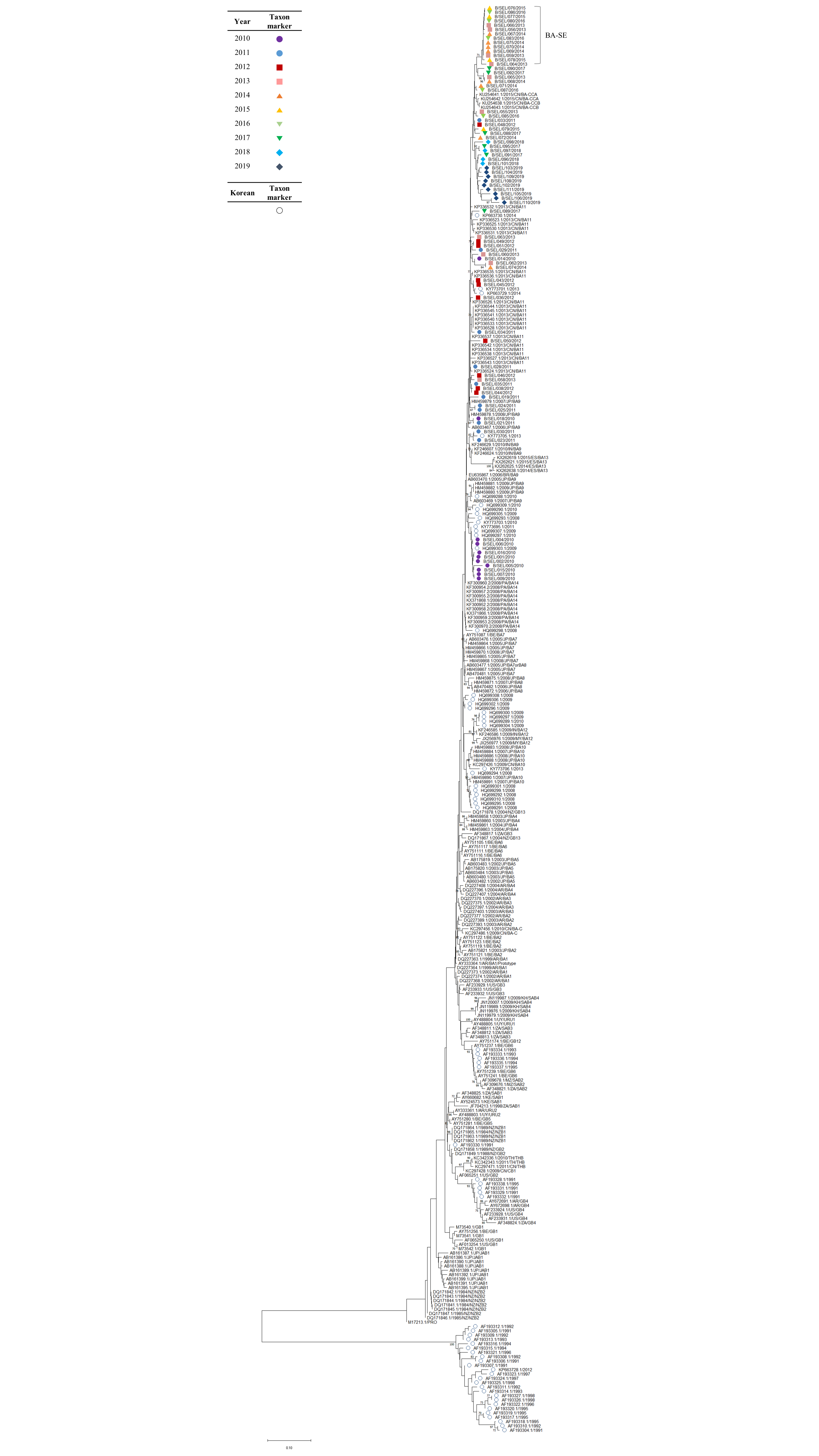

Supplement: S3 Fig — The NJ phylogenetic trees using the partial G gene sequence of Seoul RSV-A (S2 Fig) and RSV-B (S3 Fig), with their corresponding reference sequences. The NJ trees were created using maximum composition likelihood, and 1,000 bootstrap replicates by Mega X. Only bootstrap values ≥ 70% are shown for each tree. Strains are labeled with taxon markers of different colors and shapes depending on the collected year, as described in the table on the left-upper side of the tree. The Korean reference sequences (n = 67) were marked in blank black circles. (TIF) [file pone.0283873.s005.tif]

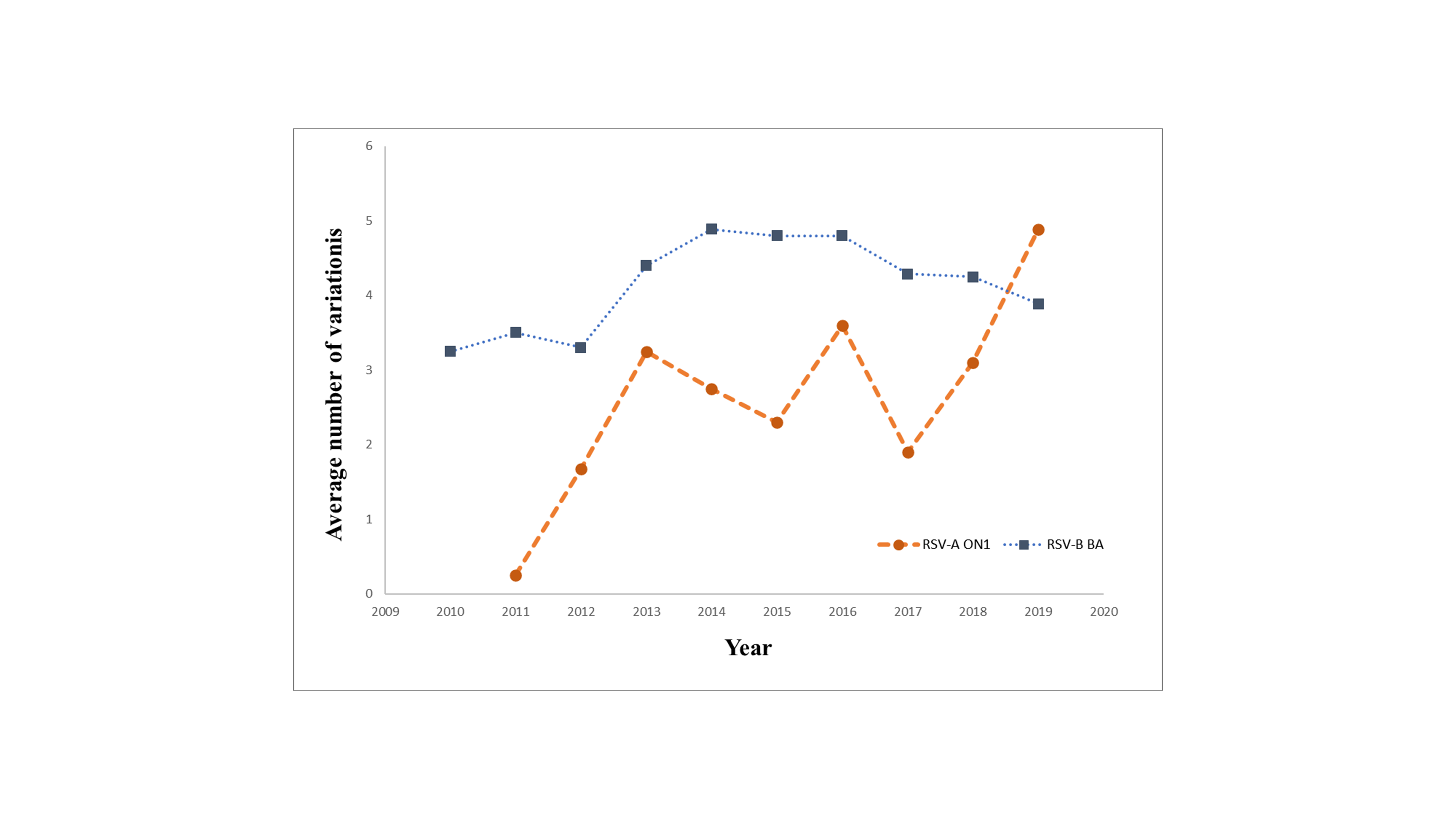

Supplement: S4 Fig — The number of variations in each sequence of Seoul RSV strains was determined compared to the reference sequences, prototype ON1 strain ON67-1210A and BA strain BA4128/99B, respectively. (TIF) [file pone.0283873.s006.tif]
